# Gut Microbiota Dysbiosis and Increased Plasma LPS and TMAO Levels in Patients With Preeclampsia

**DOI:** 10.3389/fcimb.2019.00409

**Published:** 2019-12-03

**Authors:** Jing Wang, Xunke Gu, Jing Yang, Yuan Wei, Yangyu Zhao

**Affiliations:** Department of Obstetrics and Gynecology, Peking University Third Hospital, Beijing, China

**Keywords:** gut microbiota dysbiosis, preeclampsia, lipopolysaccharide (LPS), trimethylamine-N-oxide concentration (TMAO), inflammation

## Abstract

**Objective:** To characterize the gut microbiota in patients with preeclampsia (PE) compared with healthy controls.

**Methods:** We analyzed and compared the microbiota communities in the feces of 48 PE patients with 48 age-, gestational weeks-, and pre-pregnancy body mass index-matched healthy controls using 16S rRNA gene sequencing, and also we tested fecal and plasma lipopolysaccharide (LPS) and plasma trimethylamine-N-oxide (TMAO) concentration levels in the two groups.

**Results:** Compared with the control group, microbial alpha diversity was lower in the PE group, but there was no statistically significant difference between the two groups. At the phylum level, Firmicutes (51.64% PE vs. 59.62% Control, *P* < 0.05), Bacteroidetes (40.51% PE vs. 34.81% Control, *P*< 0.05), Proteobacteria (4.51% PE vs. 2.56% Control, *P* < 0.05), and Actinobacteria (2.90% PE vs. 1.77% Control, *P* < 0.05), exhibited significant differences between the PE group and the control group. LEfSe analysis found 17 differentially abundant taxa between the two groups. PICRUSt analysis found that in the KEGG pathways, the microbial gene functions related to LPS biosynthesis were higher in the fecal microbiome of the PE group. The fecal and plasma LPS concentrations and plasma TMAO concentrations of PE patients were higher than those of the healthy controls.

**Conclusion:** PE patients had gut microbiota dysbiosis and increased plasma LPS and TMAO levels, which will lead to a better understanding of the relationship between the gut microbiota and PE.

## Introduction

Preeclampsia (PE) is a pregnancy-specific, complex and multisystem disorder characterized by hypertension and proteinuria after 20 weeks' gestation, and is one of the major causes of maternal and perinatal morbidity and mortality worldwide, affecting 3–8% of all pregnancies in the world (Plaks et al., 2013). Studies have found that early diagnosis and treatment were useful to improve the prognosis of PE (Roberge et al., 2012). Nowadays, known etiology of PE include inadequate remodeling of spiral arteries, oxidative stress, maternal vascular endothelial dysfunction and an exaggerated inflammatory response to pregnancy (Cudihy and Lee, 2009). However, the specific pathogenesis of PE remains unclear.

Gut microbiota, a complex and huge community of microorganism species living in the digestive tracts, play an important role in the host's metabolism, immunity and nutrition absorption (Viennois and Chassaing, 2018). Recent studies have found that gut microbiome dysbiosis could result in intestinal barrier disorder and bacterial translocation which would trigger a state of persistent systemic inflammation, leading to the pathogenesis and the development of diseases, such as obesity, type 2 diabetes, atherosclerosis, hypertension, and chronic kidney diseases (Andersen et al., 2017; Jonsson and Bäckhed, 2017; Li et al., 2017). Moreover, there are a number of other studies that link microbial-associated molecular patterns with inflammation and cardiometabolic disease. Caesar et al. (2012) found that gut-derived lipopolysaccharide (LPS) promotes the accumulation of macrophage in white adipose tissue which is associated with obesity. Karlsson et al. (2012) found that the gut metagenome may contribute to the development of symptomatic atherosclerosis by acting as a regulator of host inflammatory pathways. Jäckel et al. (2017) found that gut microbiota can regulate hepatic von Willebrand factor synthesis and arterial thrombus formation via Toll-like receptor-2. Thus, fecal microbiome-targeted strategies have proven to be a powerful tool for early diagnosis and treatment of different diseases.

Kell et al. consider that the originating cause of PE is microbial infection, and bacterial products such as LPS (also known as endotoxin), are well known as highly inflammagenic and could stimulate an innate immune response that aggravates inflammation action further (Kell and Kenny, 2016). However, only a few studies have investigated the relationship between gut microbiota dysbiosis and PE (Nordqvist et al., 2018). Previous studies found that probiotic milk (containing Lactobacillus bacteria) might reduce the risk of PE, through suppressing the Gram-negative bacterial LPS expression to reduce the overall systemic inflammation (Roberts and Escudero, 2012). However, whether the gut microbiota play an important role in the pathogenesis of PE remains largely unknown.

Besides, studies have found that gut microbiota-derived metabolites may play an important role in the pathogenesis of diseases and they could interact with the host through several pathways. Trimethylamine-N-oxide (TMAO) is one of the most important gut microbiota-derived metabolites and studies have found that elevated circulating TMAO concentration was a strong prognostic marker for adverse cardiac events and chronic kidney diseases (Koeth et al., 2013; Tang et al., 2015a,b; Kim et al., 2016). PE is characterized by hypertension and proteinuria, and we therefore, propose there may be potential correlations between TMAO and PE.

In this study, we analyzed and compared the gut microbiome of 48 PE patients with 48 age-, gestational weeks (GW)-, and pre-pregnancy body mass index (BMI)-matched healthy controls using 16S rRNA gene sequencing. We also compared fecal and plasma LPS and plasma TMAO levels between the two groups, in order to investigate the relationship between fecal microbiota, LPS, TMAO, and PE.

## Materials and Methods

### Subject

This study was conducted at Peking University Third Hospital from January 2018 to December 2018, and was reviewed and approved by the ethics review board of Peking University Third Hospital (2017-256-01). Signed consents were acquired from all subjects on enrolment for the use of their data and samples for scientific purposes.

The inclusion criteria for PE patients were women who matched the diagnostic criteria of the American College of Obstetricians and Gynecologists for PE (Roberts et al., 2013), including blood pressure ≥140/90 mmHg in 2 consecutive measurements at least 4 h apart, and proteinuria ≥300 mg or, in the absence of proteinuria, any of the following conditions: thrombocytopenia, renal insufficiency, impaired liver function, pulmonary edema or cerebral or visual symptoms. Exclusion criteria were: (1) multiple pregnancies; (2) diabetes, chronic hypertension, and renal diseases or other pregnancy complications before pregnancy; (3) use of antibiotics, glucocorticoids, immunosuppressive drugs within 1 month by the time of stool and plasma sample collection.

A total of 96 singleton pregnancy patients were enrolled in this study. Stool samples from 48 PE patients and 48 age-, GW-, and pre-pregnancy BMI-matched healthy controls were collected for analysis of gut microbiota and fecal LPS concentrations. Plasma samples from 30 PE patients and 30 matched healthy controls were collected for testing plasma LPS and TMAO concentrations. The information of maternal age, parity, height, body weight, GW, and urine protein amount were recorded. Pre-pregnancy BMI was derived as the weight (kg) divided by the square of the height (meters).

### Sample Collection and 16S rRNA Sequencing

Fecal samples were collected with Sarstedt feces collection containers (SARSTEDT, Nümbrecht, Germany), a 76 × 20 mm tube with a screw cap attached scoop, each of which was preloaded with 5 mL RNAlater Stabilization Solution (QIAGEN Inc.). Samples were collected in tubes by the participants and then frozen at −20°C. The samples were transferred to the laboratory on dry ice within 24 h of collection and stored at −80°C until DNA extraction.

Total fecal DNA was extracted using CTAB/SDS method (William et al., 2012). Universal primers (515F and 806R) linked with indices and sequencing adaptors were used to amplify the V4 region of the 16S rRNA gene. All PCR reactions were carried out with Phusion® High-Fidelity PCR Master Mix (New England Biolabs). After PCR products mixing and purification, sequencing libraries were generated using Ion Plus Fragment Library Kit 48 rxns (Thermo Scientific) following the manufacturer's recommendations. At last, the library was sequenced on an Ion S5TM XL platform and 400/600 bp single-end reads were generated.

After quality filtering on the raw reads according to the Cutadapt quality controlled process (V1.9.1, http://cutadapt.readthedocs.io/en/stable/) and removing the chimera sequences using UCHIME algorithm (UCHIME Algorithm, http://www.drive5.com/usearch/manual/uchime_algo.html), the clean reads were finally obtained. Sequences analysis were performed by Uparse software (Uparse v7.0.1001 http://drive5.com/uparse/). Sequences with ≥97% similarity were assigned to the same OTUs. The Silva Database (https://www.arb-silva.de/) was used based on Mothur algorithm (Schloss et al., 2009) to annotate taxonomic information.

Subsequent analysis of alpha diversity (Shannon and Simpson diversity indices, et al.) and beta diversity on weighted UniFrac was performed basing on this output normalized data and they calculated with QIIME (Version1.7.0) (Caporaso et al., 2010) and displayed with R software (Version 2.15.3). Based on the UniFrac phylogenetic distance, significant test for the clustering of samples in the study was carried out by one-way analysis of similarities (ANOSIM).

Principal Coordinate Analysis (PCoA) (Lozupone and Knight, 2005) was performed to get principal coordinates and visualize from complex, multidimensional data. A distance matrix of weighted UniFrac among samples obtained was transformed to a new set of orthogonal axes, by which the maximum variation factor is demonstrated by the first principal coordinate, and the second maximum one by the second principal coordinate, and so on. PCoA analysis was displayed by WGCNA package, stat packages, and ggplot2 package in R software (Version 2.15.3).

LEfSe analysis was applied to identify differentially abundant bacterial taxa among groups. Only those taxa that obtained a log linear discriminant analysis (LDA) score >3.5 were ultimately considered. Phylogenetic Investigation of Communities by Reconstruction of Unobserved States (PICRUSt) (Langille et al., 2013) provided a number of scripts that could be useful for analyzing both 16S rRNA gene relative abundances and the predicted metabolic data. Closed reference OTU picking against the Greengenes 13.5 database was conducted at 97% sequence similarity using the QIIME and OTU tables, and the data were normalized by single rarefaction. The resulting Biom-formatted table was normalized according to known/predicted 16S rRNA gene copy numbers, and the metagenomes were aligned to the Kyoto Encyclopedia of Genes and Genomes (KEGG) bioinformatics database. The data were analyzed statistically using STAMP, which allows data filtering and the application of different statistical tests. A two sided Welch's *t*-test was used to identify significantly different metabolic pathways in the microbiota of the two groups. *P* < 0.05 was considered to be statistically significant.

### Quantification of Fecal and Plasma LPS Concentrations

Briefly, 5 mL of RNAlater Stabilization Solution was added to 2 g feces, vortexed and centrifuged (10 min, 10,000 g, 4°C) twice. Total supernatant was filtered with 0.45 μm filter (Millipore, SLHV033RB) and then with 0.22 μm filter (Millipore, SLGP033RB). Total plasma and fecal protein concentration was analyzed through the Pierce BCA Protein assay kit. Plasma and fecal LPS levels were measured using LPS RIA Kit (Sinoukbio, Beijing, China) according to the manufacturer's protocol. Briefly, add samples, standards, antibodies and ^125^I-LPS, then vortex and incubate for 24 h at 4°C. Then add Goat Anti-Rabbit IgG serum and Normal Rabbit Serum, vortex and incubate at room temperature for 15 min. Finally, add RIA buffer, vortex and centrifuge for 20 min at 3,500 rpm, aspirate off the supernatant, and count assay tubes for calculation of results.

### Quantification of Plasma TMAO Concentrations

Plasma aliquots were stored at −80°C prior to analysis. The plasma TMAO concentrations were tested and quantified using stable isotope dilution liquid chromatography tandem mass spectrometry (Ke et al., 2018). First, a mixture with 20 μl sample and 80 μl of the internal standard mixture (10 μM d9-TMAO in methanol) was vortexed for 1 min at 4–8°C and incubated for 4 h at −80°C to precipitate protein. Then after centrifugation at 20,000 g at 4°C for 10 min, we got the supernatant, which was injected into a silica column (2.0 × 150 mm, Luna 5 u Silica 100 A; Cat. No. 00F-4274-B0, Phenomenex, Torrance, CA) at a flow rate of 0.4 ml/min using a LC-20 CE Shimadzu pump system, and a SIL-20AXR autosampler coupled to an API 5500Q-TRAP mass spectrometer (AB SCIEX, Framingham, MA). A discontinuous gradient was generated to resolve the analytes by mixing solvent A (0.1% propanoic acid in water) with solvent B (0.1% acetic acid in methanol) at different ratios starting from 2% B linearly to 95% B over 5.0 min, then holding for 1.0 min, and then reducing back to 2% B. Finally, we monitored the analytes using electrospray ionization in positive-ion mode with multiple reaction monitoring (MRM) of precursors and characteristic production transitions of TMAO at m/z 76 → 58, d9-TMAO at m/z 85 → 66, respectively. The stable isotope-labeled internal standard was purchased from Cambridge Isotope Laboratories, Inc. (DLM4779-1, Andover, MA, USA).

### Statistical Analysis

The SPSS (ver. 21.0, SPSS Inc., Chicago, IL, USA) was used for the statistical analysis. Mean (standard deviation) was used to express measurement data that obeyed a normal distribution, while the median (interquartile range) was used to express measurement data of skewed distribution. Student's *t*-test was used for quantitative variables for comparisons between the two groups. *P* < 0.05 was considered to be statistically significant.

## Results

### The Basic Characteristics of the PE Group and the Healthy Control Group

The baseline characteristics of the PE group and the control group were summarized in Table 1. No differences in age, GW and BMI were detected between the two groups (Table 1). The 24 h urine protein amount of PE group was 2.6 (1.0, 6.1) g.

**Table 1:** Clinical characteristics of the PE group and the control group.

	**PE group (*n* = 48)**	**Control group (*n* = 48)**	***P*-value**
Age, years	32.5 ± 4.7	32.8 ± 4.2	0.742
Onset gestational age, weeks	31.1 ± 5.2	32.2 ± 4.9	0.289
BMI, kg/m^2^	23.5 ± 2.6	22.6 ± 2.3	0.076
24 h urine protein amount, g	2.6 (1.0, 6.1)	N/A	N/A

*BMI, Body mass index*.

### Gut Microbiota in the PE Group and the Healthy Control Group

**Figure 1:**
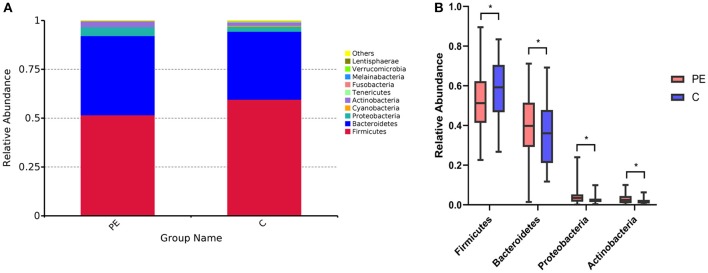
Gut microbiota in the PE group and the control group. **(A)** Difference of gut microbial community at the phylum level in the PE group and the healthy group. **(B)** The significantly different genera (Firmicutes, Bacteroidetes, Proteobacterias, and Actinobacteria) at the phylum level in the two groups. Red and blue boxes represent PE patients and the controls, respectively. The boxes represent the interquartile range (IQR) between first and third quartiles and the line inside represents the median. *P < 0.05.

A total of 96 fecal samples were obtained for sequencing. At the phylum level the majority of the OTUs were found to belong to Firmicutes (51.64% PE vs. 59.62% Control, *P* < 0.05). Bacteroidetes was the next most abundant (40.51% PE vs. 34.81% Control, *P* < 0.05) followed by Proteobacterias (4.51% PE vs. 2.56% Control, *P* < 0.05) and Actinobacteria (2.90% PE vs. 1.77% Control, *P* < 0.05), with statistically significant differences between the two groups. The remaining bacterial population belonged to the other phyla (Fusobacteria, Verrucomicrobia, Tenericutes, Cyanobacteria, and Melainabacteria), which had a relative abundance lower than 1% in the two groups (Figure 1).

### Alpha and Beta Diversity Between the PE Group and the Control Group

**Figure 2:**
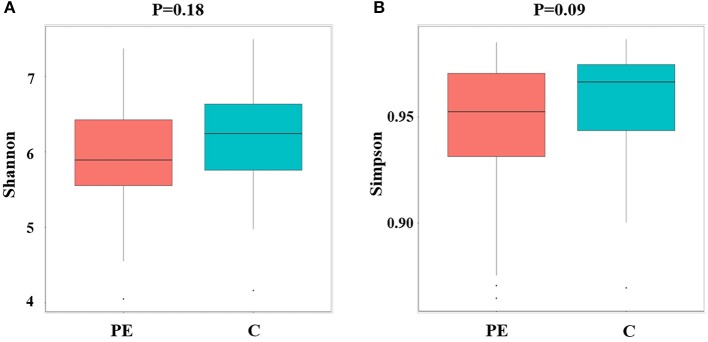
Microbial alpha diversity between the PE group and the control group. **(A)** Microbial alpha diversity (Shannon index) between the PE group and the control group. **(B)** Microbial alpha diversity (Simpson index) between the PE group and the control group.

**Figure 3:**
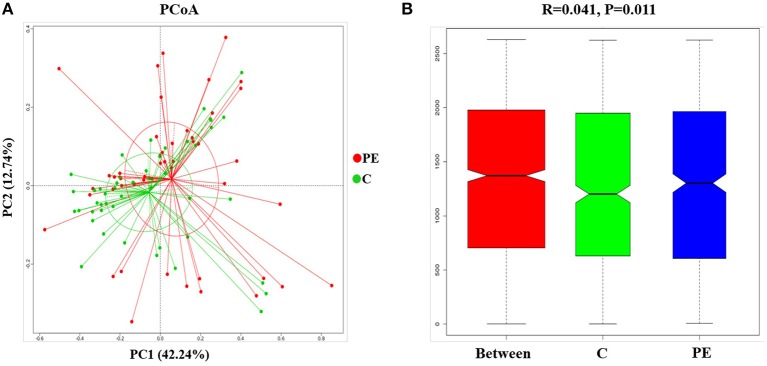
Principal coordinates analysis (PCoA) and ANOSIM between the PE group and the control group. **(A)** PCoA of Weighted UniFrac distance showed a separation of the microbiota in the PE group and the control group. PC1 scores explained 42.24% and PC2 explained 12.7% of total variations. **(B)** ANOSIM, analyses of similarities. There is a significant difference between two groups of samples (*P* < 0.05).

Compared with the healthy control group, microbial alpha diversity was lower in the PE group, as calculated by the Shannon diversity index (*p* = 0.18) (Figure 2A) and Simpson diversity index (*p* = 0.09) (Figure 2B), with no statistically significant differences. PCoA plot based on weighted UniFrac distance analysis was used to evaluate beta diversity. As shown in Figure 3A, an apparent clustering pattern was identified for the red and green points, which represented the gut microbial samples from the PE group and the control group, respectively, and a separation between the two groups could be observed from PC1 and PC2 scores that accounted for 42.24% and 12.7% of total variations. ANOSIM analysis suggested that the bacterial microflora composition of the two groups was significantly different (*P* = 0.011) (Figure 3B).

### Bacterial Taxa Differences Between the PE Group and the Healthy Control Group

**Figure 4:**
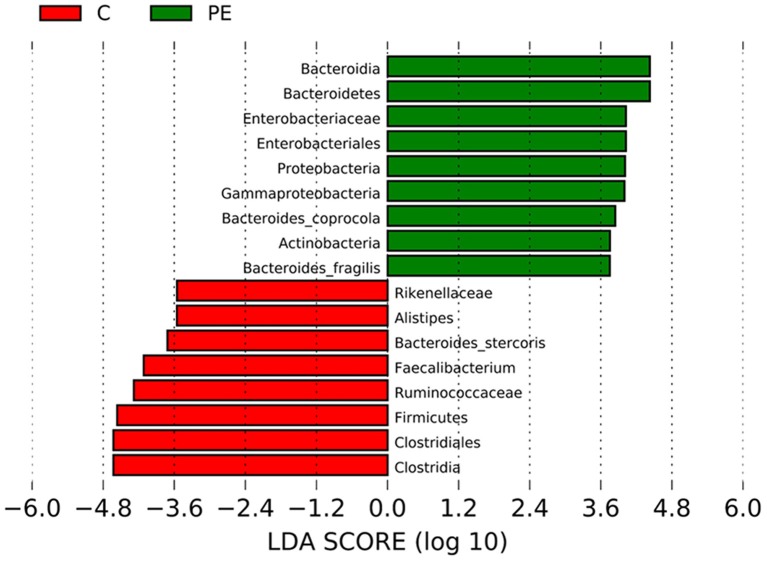
Bacterial taxa differences between the PE group and the control group using LEfSe analysis. LEfSe analysis revealed the significant bacterial differences in fecal microbiota between the PE group and the control group. The LDA scores (log10) >3.5. Green and red colors represent an increase and decrease of abundance in the PE group, respectively.

LEfSe analysis was applied to investigate the biomarkers among the two groups. We found 17 differentially abundant taxa between the two groups, all of which had a log LDA score>3.5. The relative abundances of the genera Firmicutes, Clostridia, Clostridiales, Ruminococcaceae, Rikenellaceae, Faecalibacterium, Alistipes, and Bacteroides_stercoris were lower in the PE group than those in the healthy control group, while the relative abundances of genera Bacteroidetes, Proteobacteria, Actinobacteria, Bacteroidia, Gammaproteobacteria, Enterobacteriales, Enterobacteriaceae, Bacteroides_coprocola, and Bacteroides_fragilis were higher in the PE group than those in the healthy control group. Results are presented with green and red colors indicating an increase and decrease of abundance in the PE group, respectively (Figure 4).

### Predictive Function Analysis

**Figure 5:**
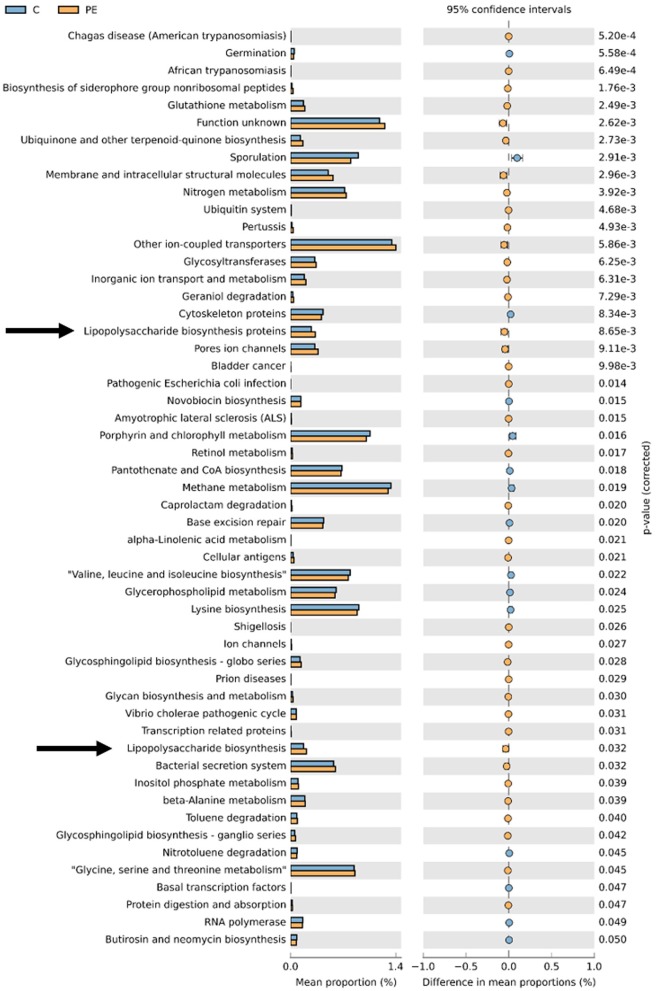
PICRUSt analysis in the KEGG pathways. Functional predictions for the fecal microbiome of the PE group and the control group. Significant KEGG pathways at level 3 for the fecal microbiome of the PE group and the healthy control group were identified by STAMP software. Arrows were over-represented LPS biosynthesis in the gut microbiome of the PE group. PICRUSt, Phylogenetic Investigation of Communities by Reconstruction of Unobserved States; KEGG, Kyoto Encyclopedia of Genes and Genomes.

PICRUSt based on closed-reference OTU was used to predict the abundances of functional categories. In the KEGG pathways, the microbial gene functions related to LPS biosynthesis were much higher in the fecal microbiome of the PE group (*P* < 0.05, Figure 5).

### Increased Fecal and Plasma LPS Levels in PE Patients

**Figure 6:**
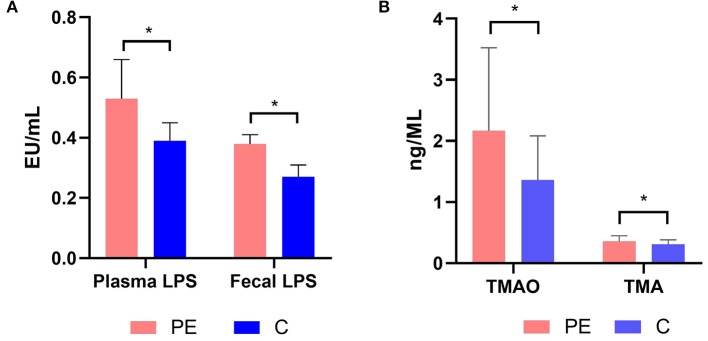
**(A)** Plasma and fecal LPS levels in the PE group and the control group. **(B)** Plasma TMAO and TMA levels in the PE group and the control group. PE patients showed significantly elevated fecal and plasma LPS concentrations. PE patients showed significantly elevated plasma TMAO and TMA concentrations. TMA, trimethylamine; TMAO, trimethylamine N-oxide. *P < 0.05.

The fecal LPS level in the PE group was 0.38 ± 0.03 EU/ml, which was higher than that of the control group (0.27 ± 0.04 EU/ml), with statistically significant difference between the two groups (*P* < 0.05). Similarly, the plasma LPS level in the PE group was 0.53 ± 0.13 EU/ml, which was higher than that of the healthy control group (0.39 ± 0.06 EU/ml). And the difference was statistically significant (*P* < 0.05) (Figure 6A).

### Increased Circulating TMAO Levels in PE Patients

The plasma TMA and TMAO concentrations were quantified using LC-MS/MS with a stable isotope as the internal standard. PE patients had higher TMA and TMAO concentrations than the healthy controls (0.36 ± 0.09, 2.17 ± 1.35 μmol/L in PE patients, while 0.31 ± 0.07, 1.36 ± 0.72 μmol/L in the healthy controls) (*P* < 0.05) (Figure 6B).

## Discussion

Gut microbiota perform multiple functions and play an important role in systemic immunity and metabolism. Healthy gut microbiota exert a vital influence on the overall health of the host (Rinninella et al., 2019). However, the relationship between the microbiome dysbiosis and PE was rarely studied. Here we report an obvious dysbiosis of gut microbiota in PE patients.

In our results, alpha diversity indices (richness and diversity) of the fecal microbiota in PE patients were lower than those of the control group, but without statistically significant difference, which was similar to Liu's study (Liu J. et al., 2017). A study of nonobese and obese Danish individuals showed that low-grade inflammation was more prevalent in patients with low gut microbiome alpha diversity (Le Chatelier et al., 2013). Koren reported that gut microbiota changed dramatically from T1 to T3, with reduced richness, which may be related to inflammation and insulin insensitivity (Koren et al., 2012).

Also, beta diversity index (structure) in patients with PE differed significantly from that of the control group. At the phylum level, the abundances of Bacteroidetes, Proteobacteria, Actinobacteria were higher, while the abundance of Firmicutes was lower in patients with PE. The members of Bacteroidetes have been reported to be associated with immunity and metabolic processes. Bacteroidetes interact with the host by glycoproteins secretion, short fatty acids imbalance, toxins production and molecular mimicry, which are involved in many diseases, such as autoimmune diseases, metabolic syndrome diseases (obesity, diabetes mellitus, atherosclerosis), and neurodegenerative disorders (Gibiino et al., 2018). A similar gut microbiota dysbiosis between Firmicutes and Bacteroidetes has been found in previous studies in association with other inflammatory disorders (Lv et al., 2016).

In our study, we found that the fecal microbiota in patients with PE showed a significant increase in Enterobacteriaceae and gamma-Proteobacteria. A recent study identified that Enterobacteriaceae species (Enterococcus Gallinarum) could induce pro-inflammatory pathways and alter gut barrier-related molecules in small intestinal tissue during translocation into internal organs (Manfredo Vieira et al., 2018). Xu Reported that gamma-Proteobacteria was higher in chronic kidney disease patients (Xu et al., 2017). In our study, we also found that the fecal microbiota in patients with PE showed a significant reduction in Clostridia, Clostridiales and Ruminococcus, which was also found in other inflammatory diseases, such as inflammatory bowel disease and Behcet's syndrome (Joossens et al., 2011; Consolandi et al., 2015; Scher et al., 2015). Interestingly, previous studies have found that several Ruminococcus species were important in maintaining gut homeostasis, particularly via the production of short-chained fatty acid (SCFAs), such as butyrate, acetate, etc. (Smith et al., 2013). Faecalibacterium, a butyrate-producing bacterium, which has anti-inflammatory activity (Haro et al., 2016), was found significantly reduced in the PE group in our study. SCFAs have been reported to influence metabolic and inflammatory responses by acting on leukocytes and endothelial cells (Vinolo et al., 2011), and recognized to have a role in the regulation of intestinal immune function. Butyrate, a key microbial metabolite, exerts an important role in the maintenance of the host immune homeostasis (Arpaia et al., 2013), showing both systemic and local immuno-modulating properties. Hu's study found that low maternal serum acetate was associated with subsequent PE, which suggested a potential role of acetate in the pathogenesis of preeclampsia (Hu et al., 2019). PE, with an imbalanced immune response in its pathogenesis, was a highly inflammagenic disease, and we speculate that microbiota dysbiosis may exert an important role in the pathogenesis of PE via abnormal expression of gut-derived metabolites.

In our study, LEfSe analysis found 17 differentially abundant taxa between the PE group and the control group, and these differentially abundant taxa may be potential biomarkers. However, they are not specific for PE, because they have been reported in other inflammatory diseases, and a broader analysis of microbiome composition may increase accuracy.

Overweight and obesity are known risk factors for PE (Bodnar et al., 2005). Previous studies have found that increased BMI has been associated with gut microbiota dysbiosis in both pregnant (Collado et al., 2008) and non-pregnant women (Gérard, 2016). In our study, PE patients had higher BMI than the control group, but there was no statistical difference between the two groups (*p* > 0.05). In order to understand the potential confounding effect of BMI on the microbiota, we used covariance analysis to adjust pre-pregnancy BMI, but we didn't find significantly changed abundant taxa between the two groups after the adjustment (Supplementary Tables 1, 2), indicating that the observed compositional differences were not strongly confounded by pre-pregnancy BMI. One possible explanation is that the average BMI of the women in this study was 23.0 ± 2.5 kg/m^2^. In future studies, in order to address the influence of obesity on the gut microbiome in women with PE, gut microbiome of obese women with and without PE should be compared and analyzed.

A functional analysis using the data obtained from the KEGG showed that LPS biosynthesis pathways were markedly over-represented in the microbiota of PE patients. Bacteroidetes have been shown to be the main contributors of LPS biosynthesis (Hevia et al., 2014). There are a number of animal models in which LPS was used experimentally to induce a condition resembling PE (Cotechini et al., 2014). Liu Y. et al. (2017) found that LPS injection in rodents could cause PE-like symptoms such as hypertension, proteinuria, adverse pregnant outcomes, and maternal inflammatory responses, both systemically and locally at the placenta. LPS injection could successfully lead to insufficient spiral artery remodeling of the placenta, and a systemic and local inflammatory response (Cotechini et al., 2014; Gong et al., 2016). LPS can activate inflammation mediated by Toll-like receptor 4 (TLR4) signaling pathway in PE (Gong et al., 2016). In PE patients, proinflammatory cytokines TNF-α and IL-6 increased in the circulation and the trophoblast cells of the placenta, while anti-inflammatory cytokines IL-10 and IL-4 decreased. Previous studies revealed that a handful of inflammatory markers, such as IL-6, IL-8, TNFα, and CRP may prove to be useful in identifying pregnant women at risk for developing PE (Black and Horowitz, 2018). This indicated that chronic peripheral and placental inflammation played a vital role in the onset of PE. In our study, we found increased plasma and fecal LPS levels in PE patients, which was consistent with Zaman's founding that serum LPS levels were higher in the PE group than those of normal pregnancy (Zaman, 2014), and we hypothesized that increased plasma and fecal LPS levels may be related to the low-grade inflammation observed in PE.

In our study, in addition to alterations in gut microbiota composition and increased fecal and plasma LPS levels, we also found that plasma TMAO concentrations were significantly higher in the PE group. TMAO, a gut microbiota-derived metabolite, has been identified as a contributing factor in the development of cardiovascular diseases and renal diseases in previous studies (Tang et al., 2013). Increased TMAO concentrations are likely linked to increased levels of TMA in the gut, which is formed by the gut microbiota from dietary sources, e.g., choline and carnitine (Fennema et al., 2016). TMAO enhances expression of inflammatory markers by promoting NF-κB activation. Sun et al. (2016) found that TMAO could significantly induce the release of inflammatory cytokines such as IL-1β and IL-18, and inhibit NO secretion in human umbilical vein endothelial cells. Koeth et al. (2013) found that TMAO could modulate cholesterol and sterol metabolism at multiple anatomic sites and processes, increasing atherosclerosis obviously, and thus he proposed a gut microbiota-TMA/TMAO-atherosclerosis pathway. Vascular “atherosclerosis-like” lesions, with lipid deposition in the walls of spiral arteries, are found in the placenta of PE patients (Staff et al., 2010). So, we speculate that TMAO may contribute to accelerated atherosclerosis which may lead to PE, and this hypothesis needs further validation.

## Limitations

Firstly, the sample size was relatively limited, and further studies with larger sample number are needed. Secondly, metagenome analysis with more sophisticated analytical capabilities will provide more detailed information to explore gut microbiota dysbiosis in PE patients closely. Thirdly, longitudinal studies which focus on the different gestational weeks of PE will be needed to reflect the relationship between gut microbiota dysbiosis and disease progression. Finally, there was no information from a diet questionnaire on nutrient intake in the study groups, and the study only described the phenomenon of gut microbiota dysbiosis and disordered gut-derived metabolite TMAO in patients with PE, without further investigation of the pathogenesis of microbiota dysbiosis underlining PE.

In conclusion, in this study we found PE patients had gut microbiota dysbiosis and increased plasma LPS and TMAO levels, which were related to inflammation and would lead to a better understanding of the relationship between the gut microbiota and PE.

## Data Availability Statement

The datasets analyzed in this manuscript are not publicly available. Requests to access the datasets should be directed to JW, Jingwang0702@126.com.

## Ethics Statement

The studies involving human participants were reviewed and approved by The ethics review board of Perking University Third Hospital. The patients/participants provided their written informed consent to participate in this study.

## Author Contributions

JW, YW, and YZ conceptualized and designed the study. JW, XG, and JY were responsible for the acquisition of data, analysis, interpretation of data, and statistical analysis. JW drafted the manuscript. YW and YZ critically revised the manuscript. YZ obtained the funding and supervised the study.

### Conflict of Interest

The authors declare that the research was conducted in the absence of any commercial or financial relationships that could be construed as a potential conflict of interest.

## Supplementary Material

Supplementary Table 1 The significantly changed taxa between the PE group and the healthy control group before and after adjustment of pre-pregnancy BMI.

Supplementary Table 2 The basic characteristics of the PE group and the healthy control group, and the significantly changed taxa between the two groups.
